# Comprehensive Profiling and Therapeutic Insights into Differentially Expressed Genes in Hepatocellular Carcinoma

**DOI:** 10.3390/cancers15235653

**Published:** 2023-11-30

**Authors:** Wesley Ladeira Caputo, Milena Cremer de Souza, Caroline Rodrigues Basso, Valber de Albuquerque Pedrosa, Fábio Rodrigues Ferreira Seiva

**Affiliations:** 1 Post Graduation Program in Experimental Pathology, State University of Londrina (UEL), Londrina 86057-970, PR, Brazil; wesley.lcaputo@uel.br (W.L.C.); milena.cremer@uenp.edu.br (M.C.d.S.); 2 Department of Chemical and Biological Sciences, Institute of Bioscience, São Paulo State University (UNESP), Botucatu 18610-034, SP, Brazil; caroline.basso@unesp.br (C.R.B.); valber.pedrosa@unesp.br (V.d.A.P.)

**Keywords:** liver cancer, drug repurposing, druggable genes, reverse expression, bioinformatics

## Abstract

**Simple Summary:**

Hepatocellular carcinoma remains crucial due to its high prevalence and the need for improved understanding and treatment options. This study utilizes extensive microarray and RNA-seq data to identify key differentially expressed genes in hepatocellular carcinoma (HCC) and FDA-approved novel druggable genes. We uncovered potential associations between metal ion exposure and tumorigenesis, as well as the relevance of kinases in HCC. Topological analysis reveals 25 hub genes and their regulatory transcription factors, while computational drug repurposing suggests several novel therapeutic candidates targeting key genes, highlighting potential avenues for future experimental assays and clinical cohorts with HCC patients.

**Abstract:**

**Background**: Drug repurposing is a strategy that complements the conventional approach of developing new drugs. Hepatocellular carcinoma (HCC) is a highly prevalent type of liver cancer, necessitating an in-depth understanding of the underlying molecular alterations for improved treatment. **Methods**: We searched for a vast array of microarray experiments in addition to RNA-seq data. Through rigorous filtering processes, we have identified highly representative differentially expressed genes (DEGs) between tumor and non-tumor liver tissues and identified a distinct class of possible new candidate drugs. **Results**: Functional enrichment analysis revealed distinct biological processes associated with metal ions, including zinc, cadmium, and copper, potentially implicating chronic metal ion exposure in tumorigenesis. Conversely, up-regulated genes are associated with mitotic events and kinase activities, aligning with the relevance of kinases in HCC. To unravel the regulatory networks governing these DEGs, we employed topological analysis methods, identifying 25 hub genes and their regulatory transcription factors. In the pursuit of potential therapeutic options, we explored drug repurposing strategies based on computational approaches, analyzing their potential to reverse the expression patterns of key genes, including AURKA, CCNB1, CDK1, RRM2, and TOP2A. Potential therapeutic chemicals are alvocidib, AT-7519, kenpaullone, PHA-793887, JNJ-7706621, danusertibe, doxorubicin and analogues, mitoxantrone, podofilox, teniposide, and amonafide. **Conclusion**: This multi-omic study offers a comprehensive view of DEGs in HCC, shedding light on potential therapeutic targets and drug repurposing opportunities.

## 1. Introduction

Precision medicine is a patient-centric approach that leverages individualized genomic information to inform drug treatment decisions, aiming to enhance clinical outcomes. In recent years, various methods have emerged to decipher and interpret multi-omics data, leading to the development of strategies for precise drug selection. These methods enable the identification of specific genetic characteristics associated with drug sensitivity or resistance while also integrating genetic markers with gene ontologies and biological networks to predict drug response on an individual level. Furthermore, by integrating multiple data sources, these approaches hold promise for optimizing drug therapies for personalized medicine [1].

Drug repositioning, or drug repurposing, involves the identification of novel uses for approved or investigational drugs beyond their original intended targets. This drug reprofiling approach offers a cost-effective and time-efficient process for drug development compared to traditional de novo drug discovery, which often involves long and expensive processes [2]. Among the numerous biological and medical applications that can benefit from drug repositioning, the field of basic and clinical oncology stands out; a quick and simple search in the Pubmed repository using the combination of “Cancer and Drug Repositioning” yields over 1100 articles in the period of 2020–2023 (accessed on 28 June 2023).

In this scenario, bioinformatics has emerged as a powerful discipline, allowing for the analysis and interpretation of large-scale biological data. With the advancement of high-throughput methodologies, such as next-generation sequencing and omics technologies, computational tools and algorithms have become crucial in the exploration of complex biological processes, disease mechanisms, and the discovery of potential therapeutic targets [1,3]. In addition, bioinformatics approaches to the study of tumor events and processes are providing insights into the underlying molecular mechanisms of the disease, which can guide the selection of potential drugs for repositioning. Just to provide further context, the combination of the terms “bioinformatics AND cancer” returned nearly 49,000 articles in PubMed between 2020 and 2023 (accessed on 28 June 2023).

Among distinct types of cancers, hepatocellular carcinoma (HCC) is the most common primary liver cancer and a leading cause of cancer-related mortality worldwide. It predominantly arises in the setting of chronic liver diseases, including hepatitis B and C virus infections, alcoholic liver disease, non-alcoholic fatty liver disease, and cirrhosis. HCC is characterized by aggressive tumor growth, high recurrence rates, and limited treatment options, necessitating a comprehensive understanding of its molecular pathogenesis and the development of effective therapeutic strategies. In this context, it is undeniable that significant progress has been made in the treatment of HCC with multiple drugs and combinations. Available drugs such as sorafenib, levantinib, regorafenib, cabozantinib, ramucirumab, nivolumab, and pembrolizumab, either alone or in combination, have proven to be effective in phase I/II studies and prolong survival in phase III randomized controlled trials. However, the limitations of these chemotherapeutics involve common side effects, including hypertension, weight loss, hand-foot skin reactions, fatigue, liver injury, and bleeding [4]. These side effects highlight the need for ongoing research and innovation in the field of oncology to address these limitations and improve the overall therapeutic experience for HCC patients.

The integration of genomic, transcriptomic, and epigenomic data has led to the identification of molecular signatures and biomarkers associated with HCC diagnosis, progression, and prognosis [5,6]. Molecular markers not only aid in early detection but also provide valuable insights into the underlying molecular mechanisms and heterogeneity of HCC, paving the way for personalized treatment approaches. To evaluate HCC through the use of bioinformatic approaches, distinct studies have proposed gene signatures for HCC [7,8,9,10,11,12]. Notably, Zhang et al. [13] identified intrinsic associations between HCC and specific genes, including *CCNA2*, *CCNB1*, *CDC20*, *CDK1*, *PTTG1*, and *TTK*, elucidating their relevance to HCC-related events. Conversely, Wang et al. [14] pointed out that the signature composed by *MARCO*, *CLEC4M*, *FCGR2B*, *LYVE1*, *TIMD4*, *STAB2*, *CFP*, *CLEC4G*, *CLEC1B*, *FCN2*, *FCN3*, and *FOXO1* reflects altered genes that may contribute to the pathogenesis of HCC, deserving a deep exploration. The disparities observed in these gene signatures are associated with different and diverse carcinogenic events. These events encompass various biological processes, such as mitotic events and stress responses to specific metal ions. Additionally, they pertain to molecular functions involving immune system responses, carbohydrate and peptide binding capacities, heme interactions, and microtubule-related activities. Moreover, these signatures implicate crucial pathways, including MAPK, FoxO, VEGF signaling, and detoxification of inorganic compounds. Consequently, it becomes evident that the ongoing debate regarding the selection of pertinent genes remains an open and intricate subject of discussion.

In part, the discrepancies found in different studies can be explained by the fact that cells from healthy (non-tumor) tissues and cells collected from tissues adjacent to the tumor (frequently referred to as adjacent-normal tissue) differ in their genetic and expression profiles [15]. Furthermore, most of the studies rely on a few microarray datasets, thus limiting their investigative outcomes. 

The efficacy of a drug to reverse cancer-associated gene expression has already been proven efficient and able to be tested in subsequent experimental procedures [16]. The success of drug repositioning for the development of anti-tumoral strategies heavily relies on the availability and utilization of bioinformatic tools and resources. These tools facilitate the analysis of diverse tumoral biological data types, such as genomic, transcriptomic, proteomic, and metabolomic data, enabling the identification of potential drug-disease associations. Herein, we used distinct bioinformatics approaches to identify hub genes and TFs that are linked to HCC and discuss possible drugs that can be repurposed in order to achieve the most effective therapeutic option for patients with liver cancer.

## 2. Materials and Methods

### 2.1. Data Mining and Processing

In the initial phase, we conducted an active and manual search in the public repository GEO (Gene Expression Omnibus, available at https://www.ncbi.nlm.nih.gov/geo/ (accessed on 10 May 2023)) to locate microarray gene expression datasets containing samples of HCC that could be compared with non-tumor tissue samples. For data processing, we utilized the R language through the R Studio platform [17]. Data processing, statistical analyses, and graphical representations were performed using R, unless explicitly stated. We followed the R script presented at https://sbc.shef.ac.uk/geo_tutorial/tutorial.nb.html# (accessed on 20 May 2023) with few adaptations to scrutinize the GEO Series (GSE) datasets; in cases where genes required normalization and resulted in a significant number of missing values (NAs or NANs), they were excluded from the study.

Our workflow was conducted based on the following inclusion criteria: (I) GSE datasets with a minimum of 10 tumor and non-tumor samples that include gene symbols or minimum information to find gene symbols; (II) the species *Homo sapiens*; and (III) access to raw data were permitted. Each dataset was individually analyzed using a univariate statistical approach with the lmFit and eBayes functions of the R package limma [18], resulting in lists of differentially expressed genes (DEGs) with log_2_ fold change (log_2_FC) and adjusted *p*-values. The threshold values log_2_FC > 2.0 and *p*-value < 0.05 indicated up-regulated DEGs, while log_2_(FC) < −2.0 and *p*-value < 0.05 indicated down-regulated DEGs. We filtered out genes without concurrent Ensembl, Entrez, and HCNG identifiers, which were gathered using hgnc [19], biomart [20], and org.Hs.eg.db [21] packages. In the cases where genes had multiple probes, we chose the probe with the highest absolute log_2_FC value. The gene distribution was visualized using Volcano plots created with the EnhancedVolcano package [22]. 

Our primary objective was to illustrate the differences between DEGs in tumor versus adjacent-tumor tissue samples and those in normal (healthy) patients versus HCC samples. For the sake of simplicity, from now on, we will refer to the “adjacent group”, the DEGs comparing adjacent tumor and tumor tissue, and to those genes that differ between healthy and tumor tissue as the “normal group”. We aimed to identify DEGs shared between these two conditions using Venn diagrams [23]. We also searched for DEGs obtained from RNA-seq analysis by comparing the TCGA database (https://www.cancer.gov/ccg/research/genome-sequencing/tcga (accessed on 1 June 2023)) and the GTEx project (https://gtexportal.org/home/ (accessed on 1 June 2023)); those genes comprise the “TCGA group”. To obtain TCGA/GTEx DEGs, we used the R script described in [24], which involved the UCSCXenaTools [25] and edgeR [26] packages. The parameters used were TCGA TARGET GTEx and TCGA Liver Cancer. For TCGA samples, we selected forPrimarySiteTCGA = “Liver,” forHistologicaltype = “Hepatocellular Carcinoma,” and forSampleType = “Primary Tumor.” For healthy tissue samples in GTEx, the parameters parastudy = “GTEX”, forPrimarysiteGTEx = “Liver,” and forPrimaryTissueGTEx = “Liver”, were chosen. The same thresholds were applied to the TCGA group: an adjusted *p*-value of 0.05 and a fold change of 2.0.

### 2.2. Functional Enrichment and Pathway Analysis

To propose a robust profile of HCC-associated DEGs, we applied a final filtering step. We selected only DEGs that appeared in at least 5 GSEs in the adjacent group and in at least 2 GSEs in the normal group. The final profile comprised the genes that were also present in the TCGA group. 

For gene enrichment analysis, we used Gene Ontology (GO) to define DEGs and their RNA or protein products and to determine the unique biological properties of transcriptomic and genomic data. The Kyoto Encyclopedia of Genes and Genomes (KEGG), Reactome pathway and Wiki pathways, which represent a collection of databases that deal with genomes, drugs, diseases, chemical materials, and biological pathways, were also consulted [13]. Enrichment analysis was carried out using enrichR [27], and the results were visualized using the ggplot2 library [28].

### 2.3. Identification of Hub Genes and Transcription Factors and Protein–Protein Interaction Network Elaboration

To propose a HCC signature, we elaborated a PPI Network and identified hub genes by analyzing the centrality of nodes (genes) using the CytoNCA plugin version 2.1.6 [29]. Four centrality measures, namely eigenvector centrality (EGC), degree centrality (DC), betweenness centrality (BC), and maximal clique centrality (MCC), were employed to identify crucial genes. The highest-ranked genes based on each centrality value were considered hub genes. Given that gene expression is regulated by specific transcription factors (TFs), which in a cancer scenario are often affected in expression, we searched for TFs associated with the hub genes in the TRRUST v.2 [30] and X2Kweb [31] databases. The relationship between TF and genes was illustrated using a Sankey diagram created with SankeyMATIC (https://sankeymatic.com/ (accessed on 1 July 2023)). 

To assess functional protein association networks and evaluate protein–protein interaction (PPI) networks, we utilized the STRING online tool (http://string-db.org, accessed on 10 July 2023). We employed the following arguments: full STRING network; active interaction sources: text mining, experiments, databases, co-expression, neighborhood, gene fusion, co-occurrence; minimum required interaction score: 0.9 (highest confidence). The resulting PPI network was visualized using Cytoscape software version 3.9.1 [32].

### 2.4. Drug Screening and the Association of Genes with Cancer Cell Events

Individual hub gene and TF expression were corroborated through the GSCA (Gene Set Cancer Analysis) database [33]. The GSCA web tool allows for the determination of the association of a set of genes with cellular events that are hallmarks of tumor cells. Next, we submitted our signature and consulted the Open Cancer Therapeutic Discovery web portal (OCTAD; http://octad.org (accessed on 10 August 2023)) [34] to search for candidate drugs that may be useful against HCC. As of September 2023, the OCTAD database comprised 19,128 samples derived from both normal and tumor tissues, sourced from a variety of reputable datasets, including GTEx, TCGA, St. Jude Hospital, MET 500, and TARGET. OCTAD performs deep-learning-based analysis, providing a drug reversal potency score that suggests complementary compounds that may be efficacious against more than 50 cancer types. The lower the score, the higher the potential the candidate drug possesses [16]. To further validate our findings, we searched for interactions between genes and chemicals by consulting the Drug–Gene Interaction Database (DGIdb; https://www.dgidb.org/ (accessed on 15 August 2023)), using the R package rDGIdb [35]. Finally, we assessed the web tool shinyDepMap (https://labsyspharm.shinyapps.io/depmap (accessed on 24 August 2023)) to check out the essentiality of genes for cancer cell survival, i.e., the growth reduction caused by knockdown/knockout (efficacy) and the selectivity of our set of genes across 423 distinct cancer cell lines [36].

## 3. Results

### 3.1. Data Mining, Filtering, and HCC—Associated Gene Identification

**Figure 1:**
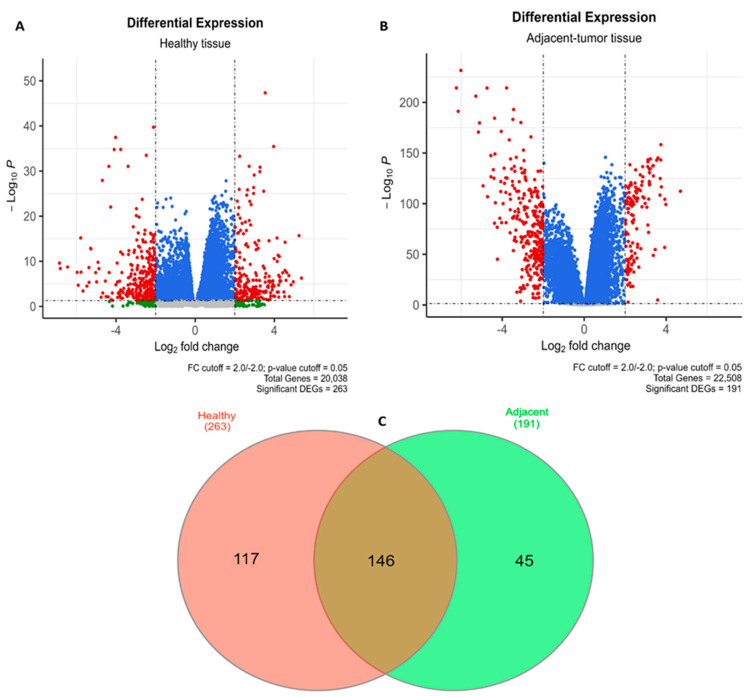
Distribution of DEGs. Volcano plots showing the distribution of DEGs found in (**A**) healthy and (**B**) adjacent tissue versus tumor tissues. Gray dots: Genes that do not fit in the FC cutoff and have *p*-value > 0.05; Blue dots: Genes that do not fit in the FC cutoff and have *p*-value < 0.05. Green dots: Genes that fit in the FC cutoff and have *p*-value > 0.05; Red dots: Genes that fit in the FC cutoff and have *p*-value < 0.05. (**C**) Venn diagram evidencing the number of shared DEGs between the two conditions.

In our initial search, we individually analyzed 17 GSEs for paired cancerous and non-cancerous adjacent tissue (adjacent group), 4 datasets for paired cancerous and non-cancerous healthy tissue (normal group), and the TCGA-LIHC/GTEx database (TCGA group), ultimately totaling 2292 HCC samples and 1361 non-tumor samples; in Table 1, the platforms, the type, and number of samples are described, and the distribution of genes per GSE is shown in Figure S1. In the adjacent group, out of a total of 22,508 genes, we filtered 191 DEGs, and for the healthy tissue samples, 263 DEGs were filtered from a universe of 20,038 genes (Figure 1A,B). An initial relevant finding was that while 146 DEGs are shared between the adjacent and normal groups, 117 and 45 genes are differentially expressed in an exclusive manner in healthy and adjacent tissues, respectively (Figure 1C). As a “quality control” of our data, we checked the mean expression of four random genes in the Liver Cancer Expression Resource database [37] to confirm there are differences between normal tissue and non-tumor adjacent tissue (Figure S2A). We also compared the fold change profile in each of the GSEs and could observe, through heat-maps, a few differences between the individual GSEs (Figure S2B). To achieve a greater degree of assertiveness in relation to DEGs, we searched for DEGs that were also described in the TCGA group; the 110 genes that constitute the intersection of the three groups were separated into 73 down-regulated and 37 up-regulated genes in HCC (Figure S3A). By heat-mapping, we made sure that log_2_FC was similar between the three conditions (Figure S3B). 

The genes *APOF*, *GPC3*, *C9*, *CLEC1B*, *CYP1A2*, *FCN3*, *HAMP*, and the gene *MT1M* were the most frequent genes found in the datasets we examined. 

To make sure that our DEGs are related to HCC, we entered the 110 genes into the DISGenet database using the R package disgenet2r [38]. As expected, the first term that returned was hepatocellular carcinoma with an extremely significant false discovery rate (FDR = 4.63 × 10^−21^), and other diseases related to the liver were also found (Table 2). 

### 3.2. Gene Ontology and Pathway Enrichment Analysis of the HCC-Associated Genes

**Figure 2:**
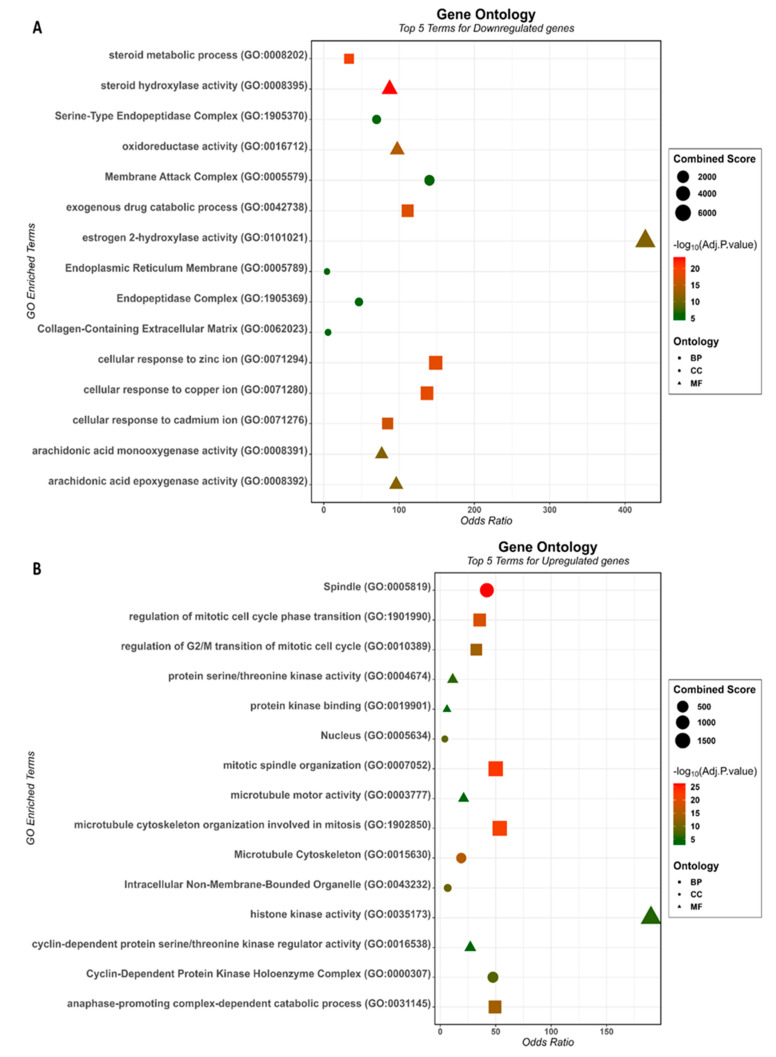
The top 5 GO terms enriched for DEGs that are (**A**) down-regulated and (**B**) up-regulated in HCC.

GO enrichment analysis, provided by the R Studio Enricher package, showed that down-regulated DEGs were significantly enriched in 64 biological processes (BP), 18 molecule functions (MF), and 6 cellular components (CC). The top five BP included “steroid metabolic process” (GO:0008202), “cellular response to zinc ion” (GO:0071294), “cellular response to copper ion” (GO:0071280), “exogenous drug catabolic process” (GO:0042738), and “cellular response to cadmium ion” (GO:0071276). The top five MF included “steroid hydroxylase activity” (GO:0008395), “oxidoreductase activity” (GO:0016712), “arachidonic acid epoxygenase activity” (GO:0008392), “estrogen 2-hydroxylase activity” (GO:0101021), and “arachidonic acid monooxygenase activity” (GO:0008391). The top five CC included “Endoplasmic Reticulum Membrane” (GO:0005789), “Membrane Attack Complex” (GO:0005579), “Collagen-Containing Extracellular Matrix” (GO:0062023), “Serine-Type Endopeptidase Complex” (GO:1905370), and “Endopeptidase Complex” (GO:1905369) (Figure 2A). Up-regulated DEGs were significantly enriched in 78 BP, 5 MF, and 13 CC. The top five terms were “mitotic spindle organization” (GO:0007052), “microtubule cytoskeleton organization involved in mitosis” (GO:1902850), “regulation of mitotic cell cycle phase transition” (GO:1901990), “anaphase-promoting complex-dependent catabolic process” (GO:0031145), and “regulation of G2/M transition of mitotic cell cycle” (GO:0010389) for BP. The top five MF were “protein serine/threonine kinase activity” (GO:0004674), “histone kinase activity” (GO:0035173), “protein kinase binding” (GO:0019901), “cyclin-dependent protein serine/threonine kinase regulator activity” (GO:0016538), and “microtubule motor activity” (GO:0003777). For GO CC, the top five terms included “Spindle” (GO:0005819), “Microtubule Cytoskeleton” (GO:0015630), “Intracellular Non-Membrane-Bounded Organelle” (GO:0043232), “Nucleus” (GO:0005634), and “Cyclin-Dependent Protein Kinase Holoenzyme Complex” (GO:0000307) (Figure 2B). 

**Figure 3:**
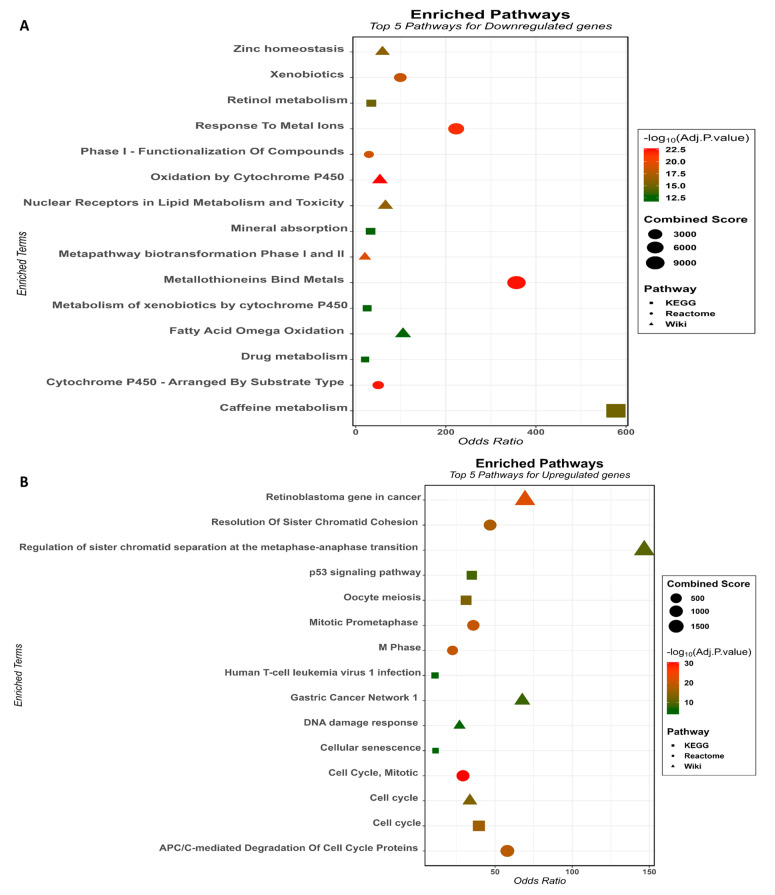
The top 5 pathways enriched for DEGs that are (**A**) down-regulated and (**B**) up-regulated in HCC.

We performed functional annotation for DEGs using three distinct pathway analyses: the Kyoto Encyclopedia of Genes and Genomes (KEGG), Reactome, and WikiPathways. Down-regulated DEGs were significantly enriched in 15 KEGG, 38 Reactome, and 29 Wiki pathways. KEGG analysis showed that down-regulated DEGs were mainly enriched in Caffeine, Retinol, Drug metabolism, “Mineral absorption”, and “Metabolism of xenobiotics by cytochrome P450”. The top five Reactome pathways were “Metallothioneins Bind Metals”, “Cytochrome P450—Arranged by Substrate Type”, “Response to Metal Ions”, “Phase I—Functionalization Of Compounds”, and “Xenobiotics”. In the Wikipathways the main terms were “Oxidation by Cytochrome P450”, “Metapathway biotransformation Phase I and II”, “Nuclear Receptors in Lipid Metabolism and Toxicity”, “Zinc homeostasis” and “Fatty Acid Omega Oxidation”. The number of significantly enriched pathways for the up-regulated DEGs was 7 (KEGG), 81 (Reactome), and 11 (WikiPathways). The top five KEGG pathways were “Cell cycle”, “Oocyte meiosis”, “p53 signaling pathway”, “Human T-cell leukemia virus 1 infection” and “Cellular senescence”. Reactome pathways were “Cell Cycle, Mitotic”, “Mitotic Prometaphase”, “M Phase”, “APC/C-mediated Degradation Of Cell Cycle Proteins” and “Resolution Of Sister Chromatid Cohesion”. The top five Wiki pathways were “Retinoblastoma gene in cancer”, “Cell cycle”, “Regulation of sister chromatid separation at the metaphase-anaphase transition”, “Gastric Cancer Network 1” and “DNA damage response”. Pathways enrichment are depicted in Figure 3A,B. 

### 3.3. Hub Genes, Transcription Factors, and the PPI Network

We used CytoNCA V2.1.6, a CytoScape plugin, for centrality analysis of the PPI network to identify crucial nodes (hub genes). The hub genes were selected based on degree centrality (DC), eigenvector centrality (EGC), betweenness centrality (BC), and maximal clique centrality (MCC) [7]. According to the centrality values, we ranked the top genes as the crucial ones; we found 25 hub genes that are described in Table S1. 

**Figure 4:**
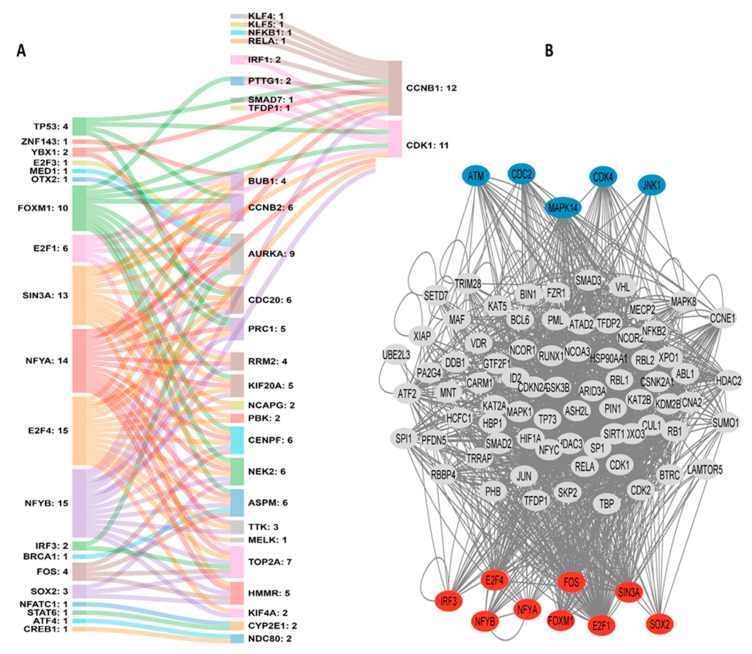
Transcription factors and Hub genes. (**A**) Sankey diagram providing insights into the relationships between TFs and genes that compose the HCC signature. (**B**) Expression kinases network, retrieved and adapted from https://maayanlab.cloud/X2K/ (accessed on 15 June 2023). Red nodes represent the top transcription factors predicted to regulate the expression of the Hub genes; gray nodes represent intermediate proteins that physically interact with the enriched TFs and connect them. Blue nodes represent the top predicted protein kinases known to phosphorylate downstream proteins.

Transcription factors (TFs) are pivotal gene expression regulators, orchestrating the intricate process by which genetic information is converted into functional molecules in living organisms. In the context of cancer, TFs play a dual role, acting as both drivers and suppressors of tumorigenesis. We searched for TFs that regulate the expression of the 25 hub genes and found 28 TFs that have been described to repress or activate a specific target gene (TRRUST database) or have a hypergeometric *p*-value lower than 0.05 (X2KWeb database). The TFs that regulate a higher number of hub genes are *E2F4* and *NFYB* (both regulating 15 genes), followed by *NFYA* (14), *SIN3A* (13), and *FOXM1* (10). The hub genes that are targets of distinct TFs were *CCNB1* (12), *CDK1* (11), *AURKA* (9), and *TOP2A* (7). Interestingly, *PTTG1* acts as both a FOXM1-regulated gene and a modulator of *CCNB1* and *CDK1* expression (Figure 4A). We also elaborated an expression kinase network displaying the inferred regulatory network predicted to regulate the hub genes. Note the protein kinases ATM, CDC2, CDK4, MAPK14, and JNK1 that act downstream in response to TF modulation and have already been associated with HCC (Figure 4B).

We explored the interconnectedness of the 53 genes that comprise our HCC signature, treating them as a cluster, and established a protein–protein interaction network employing the STRING tool. This tool serves as an invaluable resource, capable of systematically and autonomously identifying associations between proteins, including their corresponding genes, across diverse knowledge repositories, such as PUBMED, KEGG Pathways, GO terms, and Reactome, among others [39]. The PPI network was built with 52 nodes interacting via 223 edges, an average node degree of 8.58, and an enrichment *p*-value < 10^−16^ (Figure S4). 

Next, we confirmed the differential expression of hub genes (Figure S5A) and explored the expression pattern of the TFs. Except for *YBX1*, *TP53*, *TFDP1*, *STAT6*, *SIN3A*, *RELA*, *NFATC1*, *MED1*, *KLF5*, *CREB1*, and *ATF4*, the other proteins show statistically significant differences in tumor versus non-tumor hepatic tissue; specifically, the TFs *E2F1*, *FOXM1*, *PTTG1*, *BRCA1*, *E2F3*, *NFYA*, *IRF3*, *ZNF143*, *E2F4*, and *NFYB* have high expression, and conversely, the genes *NFKB1*, *SMAD7*, *IRF1*, *KLF4*, and *FOS* are down-regulated in liver cancer tissues (Figure S5B). 

### 3.4. Novel Candidate Drugs and Their Gene Expression Reversal Potential

To search for candidate-druggable genes, we submitted the 53 genes to the publicly accessible OCTAD platform (http://octad.org (accessed on 10 August 2023)) for the purpose of identifying compounds with potential utility in the treatment of HCC. Leveraging advanced deep-learning methodologies, OCTAD generates a succinct metric known as the summarized reversal gene expression score (sRGES). This metric serves as an indicator of a candidate drug’s ability to modulate gene expression profiles, specifically by either inhibiting the overexpression of particular genes or promoting the activation of genes exhibiting lower expression levels. In accordance with the recommendations provided by the platform, we considered candidate drugs with sRGES values less than −0.25 for further investigation. 

**Figure 5:**
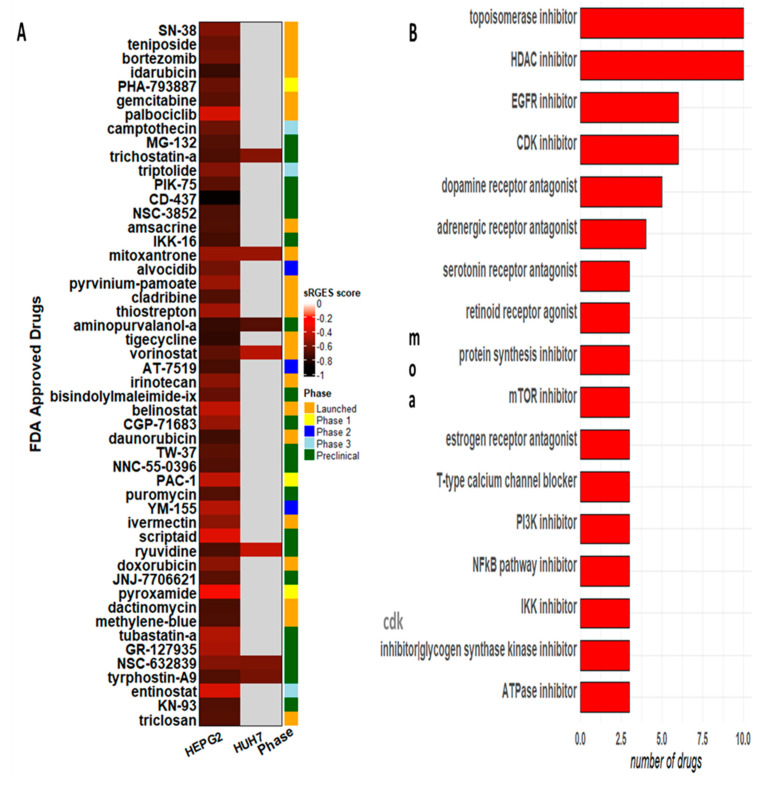
OCTAD results, showing the (**A**) top 50 ranked drugs experimented against HCC cell lines and (**B**) the main mechanism of action (moa) described by candidate drugs.

By selecting the drugs that have been tested in HCC cells only, such as Huh7.5 and HepG2, we retrieved 190 candidate compounds, which are categorized into the following experimental stages: 70 “launched”, 9 “phase 1”, 1 “phase 1/2”, 13 “phase 2”, 1 “phase 2/3”, 11 “phase 3”, 81 “preclinical stage”, and 4 drugs were withdrawn. The top 50 drugs ranked by sRGES are depicted in Figure 5A. Among the candidate compounds, we could verify a big diversity of mechanisms of action (moa), but inhibitors of HDAC, topoisomerase, CDK, EGFR, and dopamine receptor antagonists were the moa shared by at least five different chemicals (Figure 5B). From the 347 target genes retrieved from the OCTAD database, *ADRA1A*, *CDK1*, *CDK2*, *EGFR*, *GSK3B*, *HDAC1*, *HTR2A*, *HTR2C*, and *TOP2A* are target genes of at least 8 different drugs (Figure S6A). Nine of our genes are shared with the OCTAD database: *AURKA*, *CCNB1*, *CDK1*, *FOXM1*, *IRF3*, *RELA*, *RRM2*, *TOP2A*, and TP53 (Figure S6B). These genes are targets of the following drugs: danusertib and JNJ-7706621 (*AURKA*); kenpaullone (*CCNB1*); alvocidib, aminopurvalanol-a, AT-7519, CDK1-5-inhibitor, indirubin, JNJ-7706621; kenpaullone, PHA-793887 (*CDK1*); thiostrepton (*FOXM1*); piceatannol (*IRF3*); bortezomib, caffeic-acid-phenethyl-ester, pyrrolidine-dithiocarbamate, triptolide (*RELA*); cladribine, gemcitabine (*RRM2*); amonafide, amsacrine, daunurobicin, doxorubicin, idarubicin, mitoxantrone, pirarubicin, podofilox, teniposide (*TOP2A*); pifithrin-mu (*TP53).* Details about each drug are described in Table S2. 

**Figure 6:**
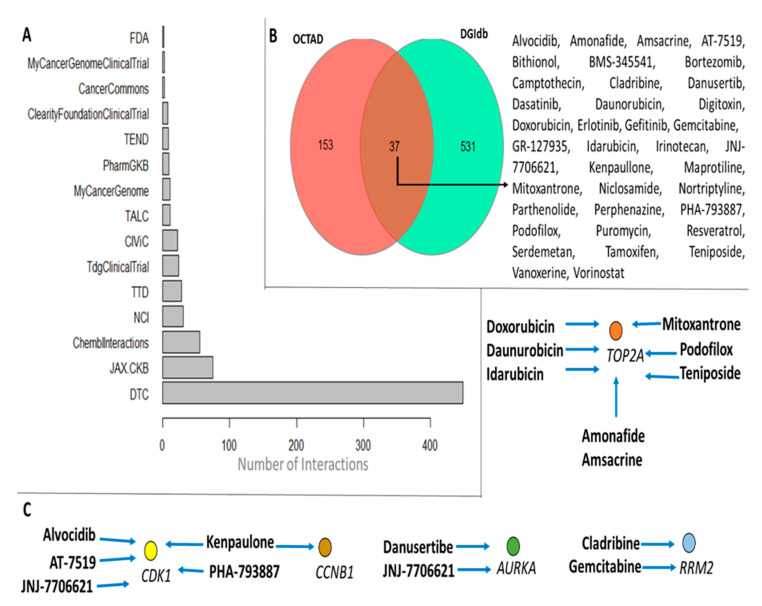
Drug and target-gene interactions: (**A**) different sources where the interactions were retrieved; (**B**) drugs shared in two distinct databases; and (**C**) druggable genes and their respective compounds. FDA: Food and Drug Administration; TEND: Trends in the exploitation of novel drug targets; TALC: Targeted Agents in Lung Cancer; CIViC: Clinical Interpretation of Variants in Cancer; NCI: NCI Cancer Gene Index; JAX-CKB: The Jackson Laboratory Clinical Knowledgebase; DTC: Drug Target Commons. Blue arrows mean that the expression pattern may be reversed after treatment.

To double-check our findings, we consulted the repository DGIdb [35]. The nine candidate-druggable genes returned 568 distinct drugs gathered from different sources (Figure 6A). The drugs are distributed as follows: 47 distinct compounds that act over AURKA, 3 over CCNB1, 48 over CDK1, 51 over RELA, 12 over RRM2, 80 TOP2A, and 377 drugs that have already been described to interact with the TP53 gene. We then opted to check out candidate drugs that are common in the two databases; 37 drugs are shared in OCTAD and DGIdb (Figure 6B). TP53 and RELA expressions did not differ in hepatic tumor tissue, so we did not consider the drugs that interact with those genes. Alvocidib, amonafide, amsacrine, AT-7519, cladribine, danusertibe, daunorubicin, doxorubicin, gemcitabine, idarubicin, JNJ-7706621, kenpaullone, mitoxantrone, PHA-793887, podofilox, and teniposide are candidate chemicals able to reverse gene expression patterns in HCC tissues; drug-gene interactions are represented in Figure 6C. The search for candidate drugs to combat HCC stands as a pivotal endeavor, fueled by the unique importance and intricate challenges posed by this type of cancer. The drug screening strategy we have put forward is a strategic pursuit designed to tackle the specific genetic or molecular traits that underlie these tumors. The drug screening we proposed can help identify and target the specific molecular intricacies of HCC and may stimulate continuous innovation within the oncology field, which can extend to the broader landscape of cancer research and therapeutics. It also offers the potential to reduce healthcare costs, streamline resource allocation, and optimize patient care. Finally, it unearths drugs that recognize and combat tumor heterogeneity, thereby taking a significant step towards tailoring treatments to the specific needs of each patient. 

**Figure 7:**
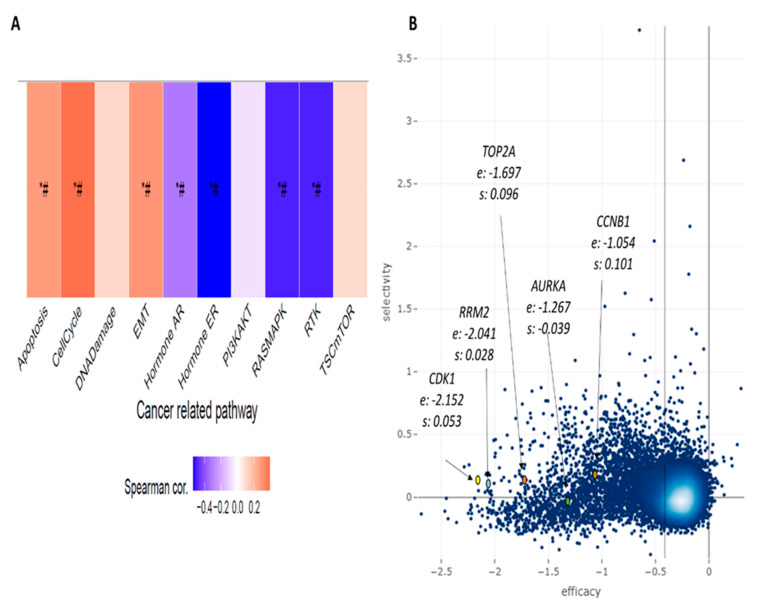
Essentiality of the five druggable genes for liver tumor cell survival: (**A**) association between GSVA score and activity of cancer-related pathways in HCC; * *p* < 0.05; # FDR < 0.05. (**B**) Efficacy (e) and selectivity (s) of genes across all cancer cell lines available at the Cancer Dependency Map (DepMap); the blue dots represent 15.847 genes evaluated. Efficacy refers to the cellular growth reduction caused by the loss of function of a specific gene.

Lastly, we verified whether the selected druggable genes are critical entities for events involved with the tumorigenesis process. Through gene set variance analysis (GSVA) and the pathway activity module, we verified some cancer-related pathways and their relationship with our genes. Apoptosis, cell cycle, and epithelial mesenchymal transition (EMT) showed a positive and significant correlation; on the other hand, the hormone AR, hormone ER, RASMAPK, and RTK pathways correlate inversely with our genes (Figure 7A). The essentiality of our genes for cell survival was evaluated by the shinyDepMap tool (https://labsyspharm.shinyapps.io/depmap (accessed on 24 August 2023)), which presents the efficacy and selectivity of specific genes regarding cell growth. Though the five genes had lower selectivity, all of them presented relative high efficacy, especially *TOP2A, RRM2,* and *CDK1* genes (Figure 7B).

## 4. Discussion

Besides being the most common primary liver malignancy, HCC also accounts for an elevated rate of morbidity and mortality. HCC is strongly associated with previous conditions and environmental risk factors, such as liver cirrhosis, HBV and HCV infections, alcohol abuse, and non-alcoholic steatohepatitis. This last one deserves special attention as it is intrinsically correlated with obesity, the 21st century epidemic [40]. Genetic alterations also contribute to HCC; thus, understanding the role played by deregulated related genes and identifying new drugs and chemicals that interact with those genes may help treat liver cancer more effectively. 

One difference between this study and other studies that have proposed gene signatures for HCC [7,9,13,14,41,42] is that we searched for and worked on a large number of microarray experiments, in addition to the well-established RNA-seq experiments gathered from TCGA and GTEx databases. After robust filtering processes, we selected only DEGs that are in common with what we called healthy, adjacent, and TCGA groups, thus resulting in a selection of highly representative genes that are differentially expressed in tumor versus non-tumor tissue. The choice of more than one filtering process may be more effective in finding DEGs in distinct types of tumors [39]. Our approach seems relevant, especially as it highlights the differences between healthy liver tissue and tissue that has already been affected to some extent by tumor cells and their microenvironment.

Some of the most frequently deregulated genes we found have been previously confirmed to be altered in HCC [10,43]. The down-regulated suppressor tumor gene *APOF* codes for Apolipoprotein F, which inhibits cholesteryl ester transfer among plasma circulating lipoproteins. In vitro and in vivo experiments showed that the reversion of *APOF* expression was effective against tumor growth, proliferation, and migration [44]. The proteoglycan *GPC3* (Glypican 3) is not normally expressed in healthy liver tissues, but the gene is up-regulated in HCC. *GPC3* is involved with Wnt signaling and Hippo pathways, both associated with liver tumor cell differentiation, survival, proliferation, migration, and invasion [45]. *CLEC1B*, encoding the CLEC-2 protein, a C-type lectin-like receptor 2, is down-regulated in HCC samples. *CLEC1B* regulates distinct signaling pathways related to immune and inflammatory responses and is inversely correlated with the proliferation and migration of HCC cells [46]. The predominantly liver-expressed cytochrome P450 1A2 (*CYP1A2*) is down-regulated in HCC. *CYP1A2* was identified as an antagonist of the hepatocyte growth factor/c-mesenchymal–epithelial transition factor (HGF/MET) signaling pathway, which is associated with tumor progression, survival, and metastasis [47]. Ficolin-3 (encoded by *FCN3*) is a protein member of the ficolin family that is down-regulated in HCC. Through pathways associated with mannose-binding lectin-associated serine proteases, ficolin-3 activates the complement system. In distinct HCC cell lines, the overexpression of *FCN3* inhibited cell proliferation and led cells to apoptosis [48]. Hepcidin (encoded by the *HAMP* gene) is a protein hormone mainly produced and secreted from the liver that, associated with ferroportin, contributes to iron homeostasis. The downregulation of *HAMP*, as we demonstrated in HCC, was also related to liver fibrosis and cirrhosis, both important risk factors for liver cancer [49]. The cysteine-rich protein Metallothionein 1M (encoded by the *MT1M* gene) participates in metal detoxification, and its overexpression was found to avoid HCC progression in vitro and in a xenograft nude mice model [50]. 

Of the 110 DEGs, most are down-regulated in HCC, while 37 are up-regulated genes. The 110 genes are closely related to liver cancer as well as other events associated with liver disease. Through GO enrichment analysis, we demonstrated that down-regulated genes are involved in distinct biological processes associated with zinc, cadmium, and cooper ions. This may not be unexpected, as chronic exposure to some metal ions has a strong association with the tumorigenesis process. Low concentrations of cadmium trigger fibrogenic and oncogenic signaling pathways in distinct HCC cell lines [51]. In vivo models also corroborate metal ion metabolism as an important event that may be involved with HCC [52]. Conversely, up-regulated gene enrichment returned distinct biological processes associated with mitotic events and molecular functions linked to kinase activities. Kinases are enzymes responsible for the ATP-dependent phosphorylation of several downstream target proteins, which in turn respond in specific patterns. Distinct kinases are known to have their expression profiles and/or activities deregulated in cancer. Excessive lactate production, a hallmark of tumor cells, induces the proliferation and metastasis of HCC cells by inhibiting adenylate kinase 2 function [53]. Furthermore, some of the actual available drugs to treat HCC are multikinase inhibitors [54]. Pathway enrichment of down-regulated genes reaffirmed the relevance of metal ions and pointed out detoxification events as relevant for HCC biology. For the genes that are up-regulated in HCC, the p53 signaling pathway and distinct events associated with cell division were enriched. Our results corroborate previous studies that evaluated distinct GSEs [10,55,56].

As can be seen in this and other studies, the number of genes with altered expression in HCC is enormous, running into hundreds. We suggest that the higher number of deregulated genes is more likely to be the consequence rather than the cause of the carcinogenic process; thus, trying to generalize about all dysregulated genes seems uninformative. One strategy is to use tools that predict degrees of centrality based on gene co-expression networks. As a mathematical model, node centrality analyses have limitations, but in biological terms, it is assumed that co-expressed genes must be co-regulated, and the degree of connectivity between genes may reflect their physiological and pathological roles [57]. We adopted 4 distinct topological analysis methods to select 25 deregulated hub genes (*ASPM*, *AURKA*, *BUB1B*, *CCNB1*, *CCNB2*, *CDC20*, *CDK1*, *CENPF*, *CYP1A2*, *CYP26A1*, *CYP2E1*, *DLGAP5*, *HMMR*, *KIF20A*, *KIF4A*, *MELK*, *NCAPG*, *NDC80*, *NEK2*, *PBK*, *PRC1*, *PTTG1*, *RRM2*, *TOP2A*, *TTK*). Transcription factors (TFs) play a pivotal role in gene regulation, orchestrating the intricate process by which genetic information is converted into functional molecules in living organisms. We found 28 TFs that regulate our hub genes and showed *E2F4*, *NFYB*, *NFYA*, *SIN3A*, and *FOXM1* to control at least 10 different genes. The overexpression of those five TFs has already been described to participate in different stages and events related to liver cancer and to have prognostic values for patients with HCC [58,59,60]. The significance of TFs in gene regulation extends across diverse biological contexts, driving advancements in our understanding of complex genetic networks, disease etiology, and potential therapeutic interventions. 

Chemotherapy remains the cornerstone of treatment for solid tumors like HCC, with various drugs and regimens under constant investigation. Notably, the drug 5-fluorouracil (5-FU), a breakthrough that emerged from research on rat hepatoma, has played a pivotal role in this field. First synthesized in 1950 by Charles Heidelberger and collaborators and approved for human trials in 1962, 5-FU undergoes metabolic transformations involving enzymes like dihydropyrimidine dehydrogenase, orotate phosphoribosyltransferase, uridine phosphorylase, and uridine kinase. Through a series of phosphorylation steps, 5-FU is converted into active metabolites, including FdUTP and FdUMP, which disrupt DNA synthesis by irreversibly inhibiting thymidylate synthase. Thymidylate synthase is essential for DNA synthesis as it converts deoxyuridine monophosphate (dUMP) into deoxythymidine monophosphate (dTMP), a basic component of pyrimidines [61,62]. Thus, thymidylate synthase inhibition exhibits a potent anti-cancer mechanism. However, 5-FU, like many chemotherapy drugs, is associated with significant systemic toxicity [63], underscoring the need for novel adjuvants to mitigate adverse effects and enhance the overall efficacy of anti-cancer treatments.

A promising strategy in therapeutic treatment involves the systematic re-utilization of drugs with well-established safety and pharmacokinetic profiles, considering that numerous drugs possess multiple targets and targets can be influenced by multiple drugs. The exploration of drug repositioning extends beyond approved drugs and encompasses a growing pool of late-stage failures that have been halted due to insufficient efficacy or safety concerns [64]. One strategy for drug repurposing is based on computational approaches, which revolve around the analysis of pre-existing data sources, including chemical structures, gene expression data, proteomic information, and electronic health records [36]. Drug repurposing offers distinct advantages such as diminished initial drug development prerequisites, financial and temporal savings, and a heightened likelihood of receiving regulatory approvals compared to the conventional de novo drug discovery route. After consulting two distinct drug-gene interaction databases, we found 18 chemicals that have been tested in HCC cells and have the potential to reverse the gene expression pattern of the protein-coding genes *AURKA*, *CCNB1*, *CDK1*, *RRM2*, and *TOP2A*. We also showed that the loss-of-function of *CDK1*, *RRM2*, and *TOP2A* has the highest efficacy in reducing cellular proliferation. 

*CDK1* (Cyclin-Dependent Kinase 1) is a critical regulator of the cell cycle, and its dysregulation has been implicated in various cancers. We found *CDK1* to be upregulated, which has been associated with tumor development and HCC progression. Five drugs may interact with *CDK1*: **(i) Alvocidib**, the cyclin-dependent kinase inhibitor, is being considered to treat acute myeloid leukemia [65]; **(ii) AT-7519**, the second-generation small molecule multi-CDK inhibitor, was experimented against glioblastoma, lung cancer, myeloma, and leukemic cells [66,67,68,69]; more recently, AT-7519 has demonstrated anti-tumoral effects against HCC [70]; **(iii) Kenpaullone**, a multiple kinase inhibitor known for its inhibitory potential of GSK3 activity, has been experimented to be effective in some neurological disorders, cystic fibrosis, ototoxicity, and preventing oxidative stress damage in cardiomyocytes [71,72,73,74]. Kenpaullone may also act upon *CCNB1*, the gene that encodes Cyclin B1. The *CCNB1* gene is significantly up-regulated in HCC, and there is a positive correlation with *CCNB1* overexpression and vascular invasion in HCC samples [75]. We did not find experimental studies regarding kenpaullone and HCC. **(iv) PHA-793887**: Few experimental studies have been conducted to certify its activity, but it was demonstrated in vitro that PHA-793887 was able to interfere with the viability of osteosarcoma cells [76]. **(v) JNJ-7706621**: a potent inhibitor of CDKs and Aurora kinase (AURKA) demonstrated effectiveness in dealing with the herpes simplex virus 1 [77].

*AURKA*, coding for Aurora A kinase, is up-regulated in HCC. *AURKA* is a mitotic regulator that is subject to several regulatory interactions and post-translational modifications, including covalent CoA modification induced by oxidative stress [78]. Besides JNJ-7706621, **danusertibe** is able to reverse the *AURKA* expression. Danusertibe exhibits inhibitory activity against all known Aurora kinases and was identified to inhibit DNA helicases [79]. Danusertibe suppressed liver tumor cell proliferation in vitro and in vivo [80]. 

Ribonucleotide reductase regulatory subunit M2 (*RRM2*) overexpression found in HCC samples increased the proliferative and migratory capabilities of Hep3B and Huh7 cells [81]. *RRM2* is the target of **cladribine** and **gemcitabine**. Some of the applications for cladribine are for treating acute myeloid leukemia, the rare Rosai–Dorfman disease, and multiple sclerosis [82,83,84]. The heterocyclic drug gemcitabine, a nucleoside analogue of deoxycytidine, acts in synergism with sorafenib to improve the chemoresistance of Huh7 cells [85]. 

*TOP2A* is the druggable gene that we found to be the target of eight distinct drugs. Among them, we showed that the anthracycline antibiotics and classic cancer chemotherapeutics **(i) doxorubicin**, **(ii) daunorubicin**, **and (iii) idarubicin** are predicted to be able to reverse *TOP2A* upregulation. Their chemical properties and differences can be consulted elsewhere [86]. Inhibition of *TOP2A* by doxorubicin contributes to suppressing the growth of sorafenib-resistant HCC tumors in vitro and in vivo [87]. At this point, it is imperative to address a key facet of HCC treatment with doxorubicin. Despite its widespread use as a cytotoxic agent, it is crucial to acknowledge the ongoing debate regarding doxorubicin’s clinical activity, with some asserting that it either lacks significant demonstrable benefits or offers minimal efficacy against HCC when used systemically [88,89]. As a potential alternative, a single-center clinical trial highlighted that transarterial chemoembolization using idarubicin exhibits a favorable safety profile, achieves high tumor response rates, and extends time to progression significantly [90,91].

Similar to doxorubicin and idarubicin, **(iv) Mitoxantrone** also has the ability to reduce the growth of chronic myeloid leukemia K562 cells by a mechanism involving proteasomal activity [92]. **(v) Podophyllotoxin (Podofilox)**, an aryltetralin cyclolignan extracted from the roots and rhizomes of *Podophyllum* species, and its derivative **(vi) teniposide** have already shown anti-tumoral properties for distinct types of neoplasia, including HCC [93,94,95]. **(vii) Amonafide**, a naphthalimide, was initially tested to treat acute myeloid leukemia and breast cancer. By intercalating DNA and blocking the binding of topoisomerases, amonafide promotes apoptotic cell death [96] and was effective against HepG2 and Huh7 cells [97]. The synthetic aminoacridine derivative **(viii) amsacrine** has a mechanism of action similar to amonafide and showed potential effects in treating malignant lymphoma and acute myeloid leukemia [98]. We found no recent study dealing with amsacrine and HCC.

## 5. Perspectives and Limitations

The concomitant administration of drugs with distinct mechanisms of action holds promise as a viable strategy for addressing neoplastic conditions. The synergy resulting from the combination of two or more drugs can yield superior therapeutic outcomes in the context of HCC, as demonstrated by prior research [99]. To cite a few examples, the co-inhibition of AURKA and HSF1 has exhibited remarkable anti-tumor efficacy against HCC cells, both in vitro and in vivo [100], while the concurrent use of danusertibe and sorafenib has been shown to produce additive effects [80]. Furthermore, pharmacologically active compounds with new chemical structures analogous to and derived from natural compounds, such as congeners of podophyllotoxin and amonafide, are possible and promising options that deserve further evaluation [94,97]. Despite the limitations commented below, our study underscores the undeniable importance of repurposing novel chemical candidates for addressing HCC. This includes the promising avenue of combining predicted drugs with established compounds in conventional chemotherapeutic regimens, thereby shedding light on innovative drug replacement strategies. 

This study encountered two relevant constraints. Firstly, bioinformatics analyses were executed without undergoing experimental validation. Secondly, empirical verification is essential for confirming the identified hub genes and drug-gene interactions. With these limitations in mind, investigations akin to ours not only broaden the spectrum of potential therapeutic approaches but also pave the way for forthcoming experimental studies.

## 6. Conclusions

Through a comprehensive evaluation and comparison of multiple datasets highlighting DEGs in HCC, we have meticulously curated a robust list of genes. This compilation comprises 25 DEGs along with their associated transcription factors. Notably, *CDK1*, *TOP2A*, and *RRM2* emerge as promising candidates for potential drug testing, either in isolation or in combination. Upon successful experimental validation of these novel therapeutic approaches, they hold the potential for clinical testing within a specific cohort of HCC patients.

## Figures and Tables

**Table 1:** Datasets manually curated to identify DEGs commonly found in HCC.

GSE	Plataform	Type	Tumor Sample	Non-Tumor Sample
102079	GPL 570	Adjacent-tissue	152	91
121248	GPL 570	Adjacent-tissue	70	37
12941	GPL 5175	Adjacent-tissue	10	10
136247	GPL 17586	Adjacent-tissue	39	30
14520	GPL 571	Adjacent-tissue	225	220
22405	GPL 10553	Adjacent-tissue	24	24
25097	GPL 10687	Adjacent-tissue	268	243
36376	GPL 10558	Adjacent-tissue	240	193
39791	GPL 10558	Adjacent-tissue	72	72
41804	GPL 570	Adjacent-tissue	20	20
45267	GPL 570	Adjacent-tissue	46	41
57957	GPL 10558	Adjacent-tissue	39	39
60502	GPL 96	Adjacent-tissue	18	18
64041	GPL 6244	Adjacent-tissue	60	60
76427	GPL 10558	Adjacent-tissue	115	52
84005	GPL 5175	Adjacent-tissue	38	38
84402	GPL 570	Adjacent-tissue	14	14
102079	GPL 570	Healthy liver	152	14
112790	GPL 570	Healthy liver	183	15
62232	GPL 570	Healthy liver	81	10
50579	GPL 14550	Healthy-liver	67	10
TCGA x GTEx	RNA-Seq	-	359	110

**Table 2:** DISGENet results showing strong association between DEGs and liver events.

Term	Ratio	FDR
Liver carcinoma	68/3593	4.63 × 10^−21^
Carcinogenesis	55/4065	3.02 × 10^−9^
Liver diseases	19/606	1.50 × 10^−7^
Chronic liver disease	10/129	3.18 × 10^−7^
Hepatocarcinogenesis	17/527	4.73 × 10^−7^
Liver neoplasms	26/1321	8.22 × 10^−7^
Malignant neoplasm of liver	20/805	1.26 × 10^−6^
Liver and Intrahepatic Biliary Tract Carcinoma	15/607	6.31 × 10^−5^

## Data Availability

The datasets reanalyzed during the current study are available in the GEO (https://www.ncbi.nlm.nih.gov/geo/ (accessed on 10 May 2023)), TCGA (https://portal.gdc.cancer.gov/ (accessed on 1 June 2023)), and DepMap (https://depmap.org/ (accessed on 24 August 2023)) repositories. The data generated during this study are included in this published article and its Supplementary Information Files.

## Supplementary Materials

The following supporting information can be downloaded at: https://www.mdpi.com/article/10.3390/cancers15235653/s1, Data S1: All genes found in GSEs of Healthy Samples. Data S2: All genes found in GSEs of Adjacent Samples. Data S3: Shared genes in details. Data S4: Transcription Factors. Figure S1: Circos-plot showing the number and distribution of genes by GSE. Figure adopted from the web tool MetaScape. Figure S2: (A) Difference in the mean expression between Normal and Adjacent non-tumor tissue (data extracted from the cancerlivER database, available at https://webs.iiitd.edu.in/raghava/cancerliver/index.html); HCC: Hepatocellular carcinoma; CCA: Cholangiocarcinoma; HA: Hepatic Adenoma. (B) Heat-maps with log2FC from Normal versus tumor tissue and Adjacent-non tumor versus tumor tissue. Figure S3: DEGs and their differential expression profile: (A) Venn diagram showing DEGs shared between Healthy, Adjacent, and TCGA groups; (B) Heat-maps of the 110 DEGs with log2FC. Figure S4: PPI network for HCC signature with minimum required interaction score of 0.9 (highest confidence). Nodes with no connections are not shown. Data obtained and adapted from the STRING database. Figure S5: Differential Expression of (A) Hub genes and (B) Transcription factors. The data are based on normalized and batch-corrected RSEM mRNA expression. Retrieved and adapted from Liu, C. J., Hu, F. F., Xia, M. X., Han, L., Zhang, Q., and Guo, A. Y. (2018), GSCALite: a web server for gene set cancer analysis, Bioinformatics 34, 3771–3772. Figure S6: Target genes found in (A) the OCTAD database and (B) shared with our DEGs. Table S1: Top genes ranked by distinct node centralities of the PPI network. Table S2: Details about drugs that can potentially reverse the expression pattern of DEGs found in HCC. Data retrieved from Pubchem (https://pubchem.ncbi.nlm.nih.gov/, accessed in September, 2023). CAS: Chemical Abstracts Service; moa: mechanism of action. * The synonyms provided here are not the only ones available in the database. Reference [101] are cited in the supplementary materials.
