# Supplementary material for: Comprehensive Profiling and Therapeutic Insights into Differentially Expressed Genes in Hepatocellular Carcinoma
Source: Cancers (Basel). 2023 Nov 30;15(23):5653. doi: 10.3390/cancers15235653 (PMC10705715; doi:10.3390/cancers15235653)
Supplement: Supplementary file 1 [file cancers-15-05653-s001.zip › SUPPLEMENTARY MATERIAL.pdf]

Comprehensive Profiling and Therapeutic Insights into Differentially Expressed Genes in Hepatocellular Carcinoma

SUPPLEMENTARY MATERIAL

Data Set	n° of Genes	Color Code
GSE102079	196	
GSE121248	118	
GSE12941	78	
GSE136247	116	
GSE14520	163	
GSE22405	18	
GSE25097	363	
GSE36376	55	
GSE39791	58	
GSE41804	281	
GSE45267	299	
GSE57957	32	
GSE60502	185	
GSE64041	1	
GSE76427	12	
GSE84002	492	
GSE84005	180	

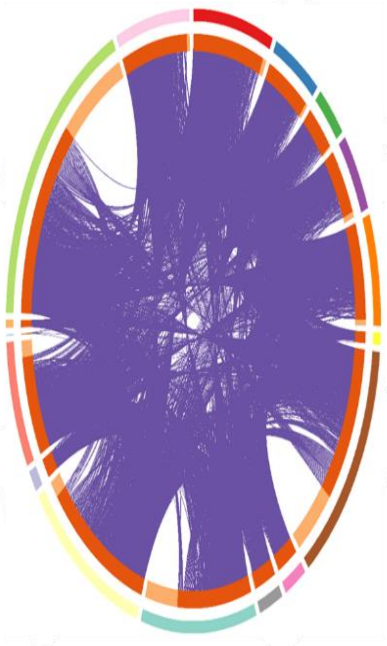

Adjacent GSEs

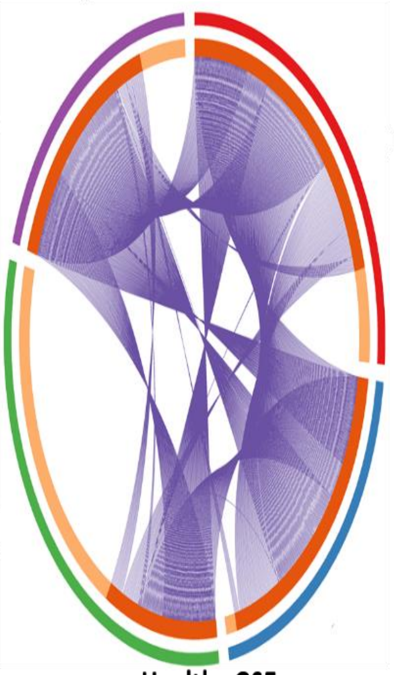

Healthy GSEs

Data Set	n° of Genes	Color Code
GSE102079	196	
GSE112790	2288	
GSE50579	354	
GSE62232	221	

Figure S1. Circos-plot showing the number and distribution of genes by GSE. Figure adopted from the web tool MetaScape.

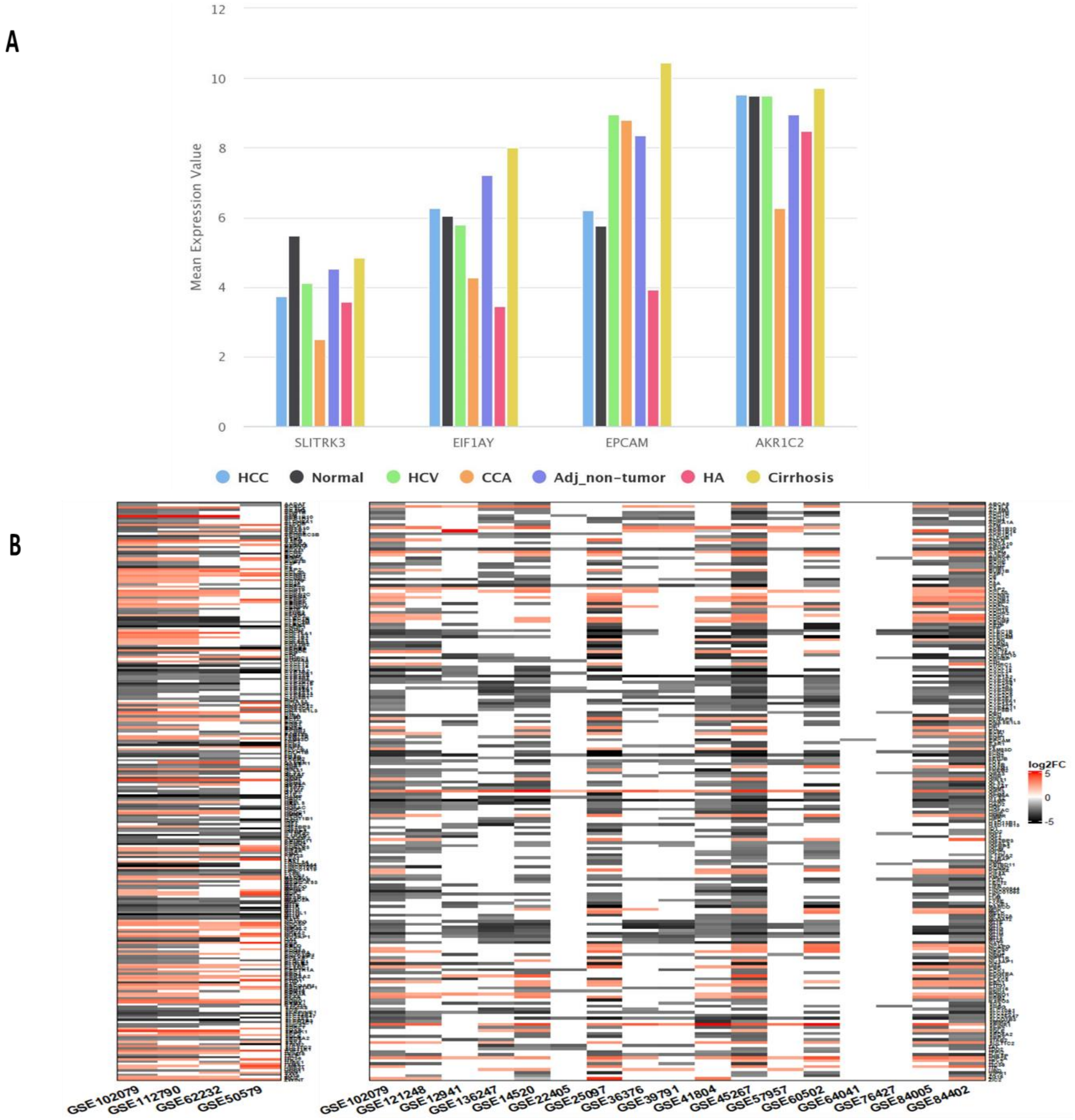

Figure S2. A) Difference in the mean expression between Normal and Adjacent non-tumor tissue (data extracted from cancerlivER database, available at https://webs.iiitd.edu.in/raghava/cancerliver/index.html); HCC: Hepatocellular carcinoma; CCA: Cholangiocarcinoma; HA: Hepatic Adenoma. **B)** Heat-maps with log_2FC from Normal versus tumor tissue, and Adjacent-non tumor versus tumor tissue.

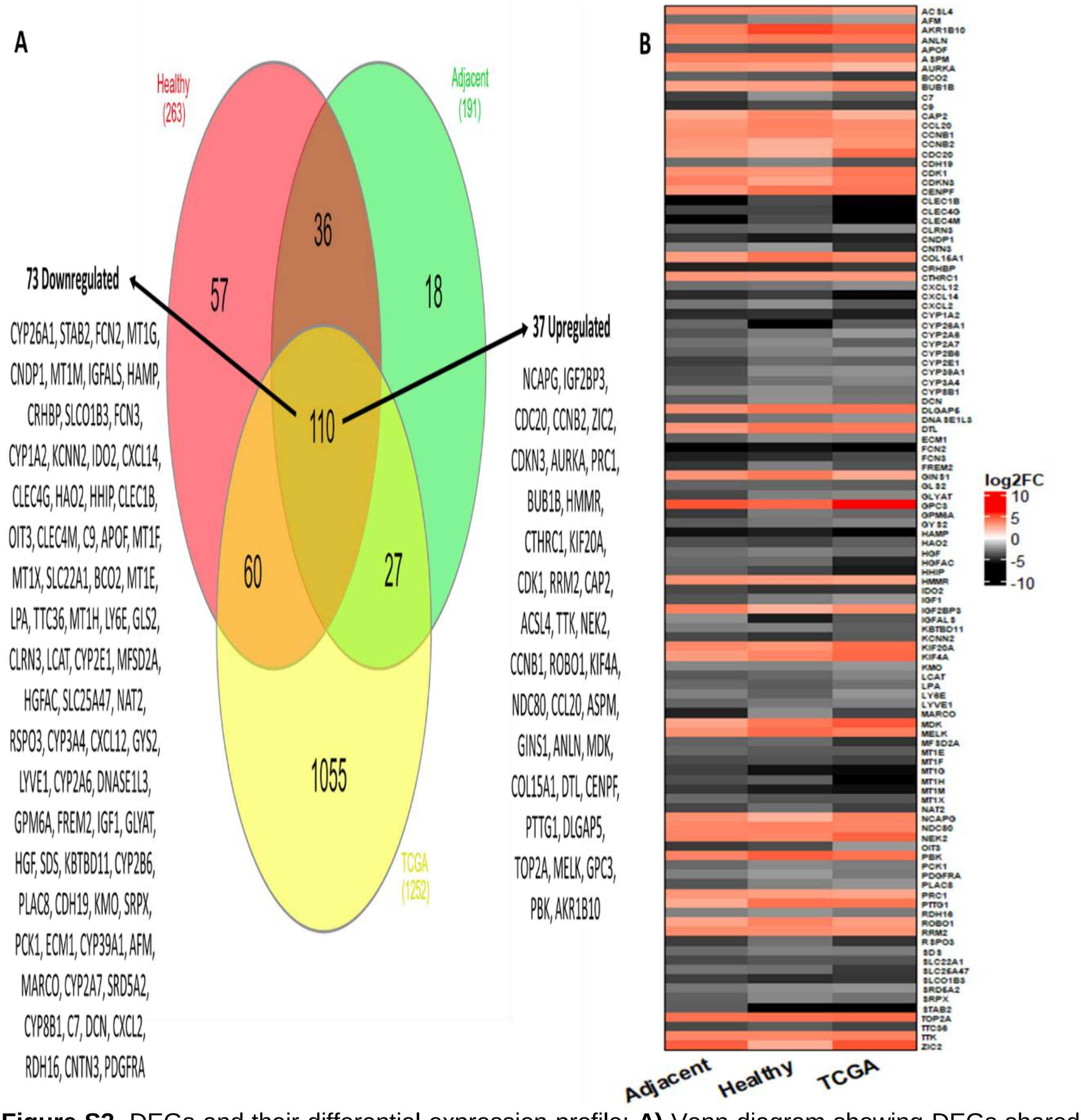

Figure S3. DEGs and their differential expression profile: **A)** Venn diagram showing DEGs shared between Healthy, Adjacent and TCGA groups; **B)** Heat-maps of the 110 DEGs with log_2FC.

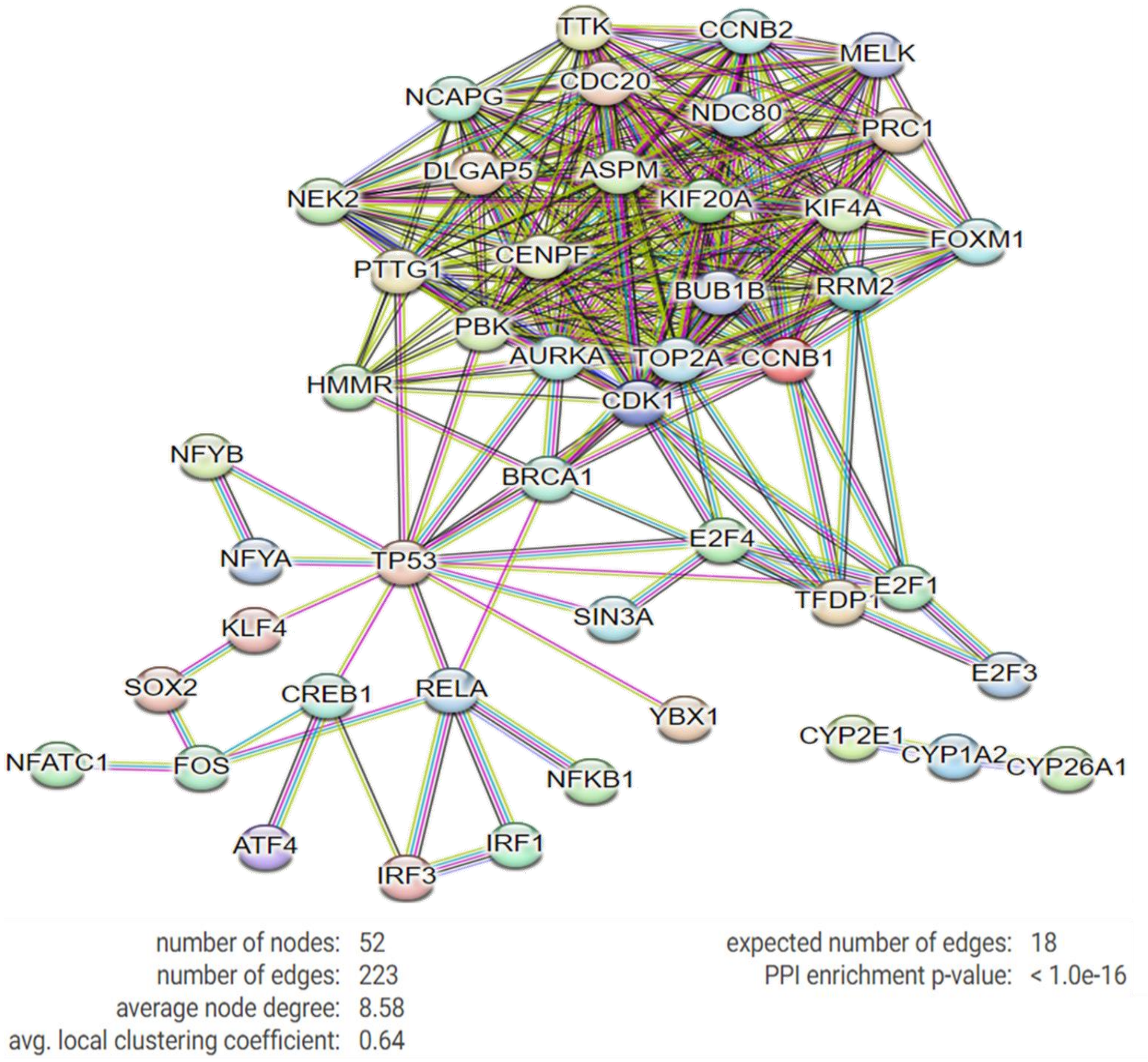

Figure S4. PPI network for HCC signature with minimum required interaction score 0.9 (highest confidence). Nodes with no connection are not show. Data obtained and adapted from STRING database.

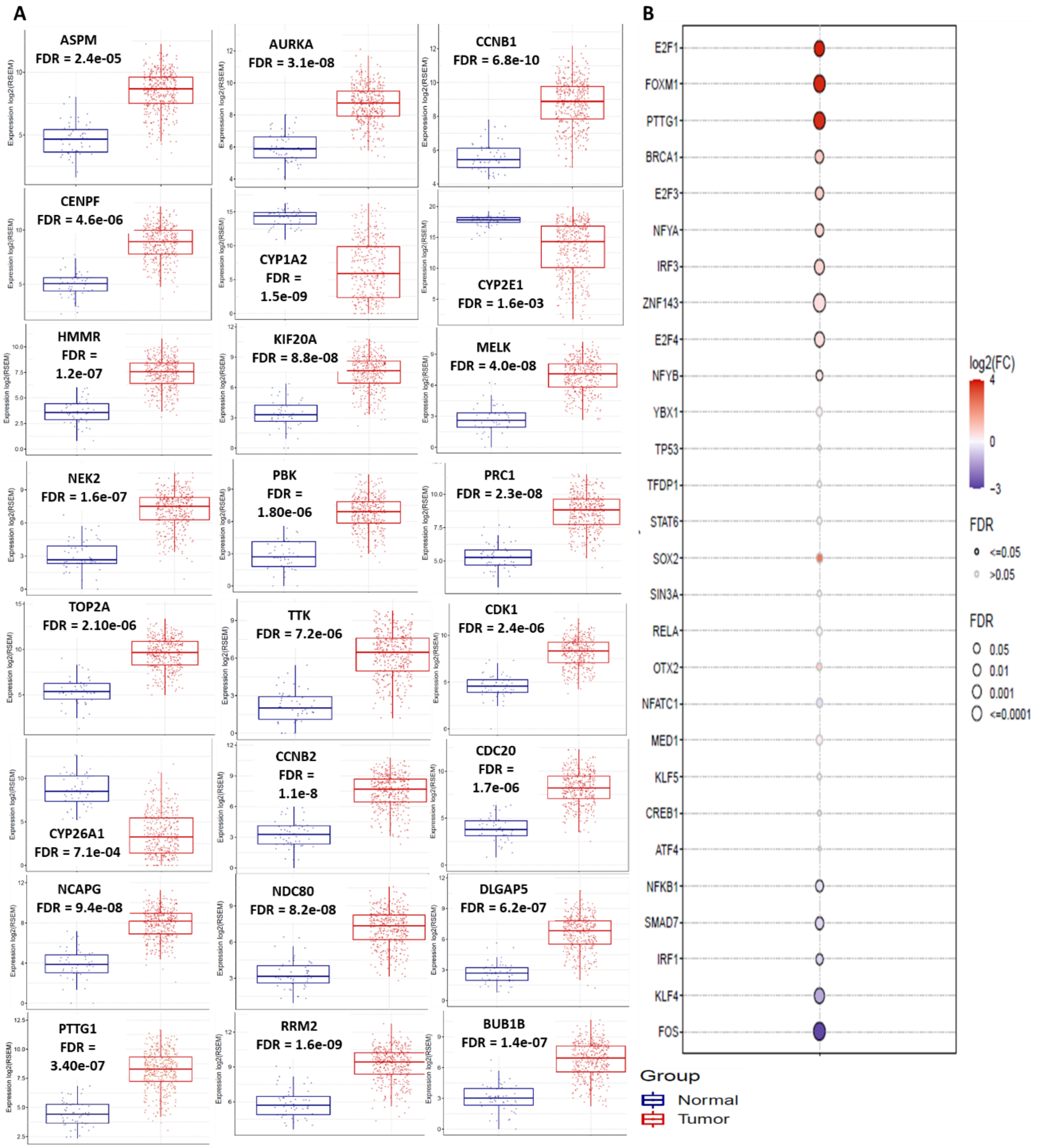

Figure S5. Differential Expression of **A)** Hub genes and **B)** Transcription factors. Data are based on normalized and batch corrected RSEM mRNA expression. Retrieved and adapted from [1]

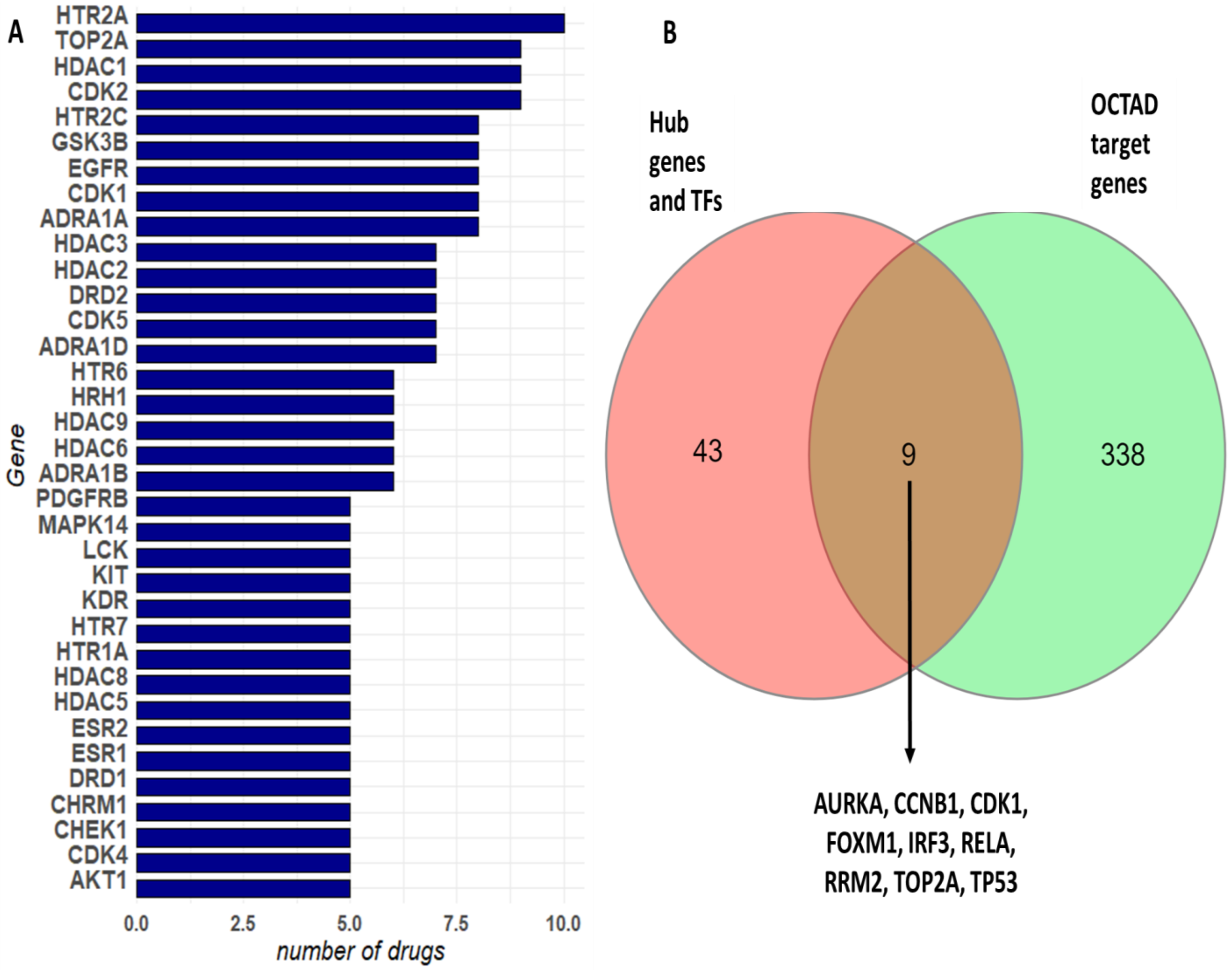

Figure S6. Target genes found in **A)** OCTAD database, and **B)** shared with our DEGs.

Table S1. Top genes ranked by distinct node centralities of the PPI network.

Centrality			
**Degree**	**Eigenvector**	**Betweenness**	**MCC**
CDK1	CDK1	HMMR	CDK1
ASPM	ASPM	CDK1	ASPM
DLGAP5	DLGAP5	ASPM	DLGAP5
PBK	PBK	CYP1A2	PBK
TOP2A	TOP2A	CENPF	TOP2A
NCAPG	KIF20A	CYP2E1	CENPF
CENPF	NCAPG	CYP26A1	NCAPG
KIF20A	AURKA	DLGAP5	AURKA
AURKA	CCNB1	PBK	KIF20A
CCNB1	CCNB2	TOP2A	PTTG1
CCNB2	CDC20		BUB1B
CDC20	CENPF		CCNB1
PTTG1	KIF4A		CCNB2
BUB1B	NDC80		CDC20
KIF4A	TTK		KIF4A
NDC80	BUB1B		NDC80
TTK	PTTG1		TTK
	MELK		MELK
	RRM2		RRM2
	PRC1		PRC1
	NEK2		MELK

Table S2. Details about drugs that can pontentially reverse the expression pattern of DEGs found in HCC.

Drug	PubChem CID	CAS number	Synonyms*	moa
Danusertib	11442891	827318-97-8	PHA-739358	Aurora kise inhibitor/ Growth factor receptor inhibitor
Alvocidib	5287969	146426-40-6	Flavopiridol	CDK inhibitor
Aminopurvalanol-A	6604931	220792-57-4	----------	CDK inhibitor / Tyrosine kise inhibitor
AT-7519	11338033	844442-38-2	----------	CDK inhibitor
Quinoxaline1	438981	56984-56-6	CDK1-5-inhibitor	CDK inhibitor / Glycogen synthase kinase inhibitor
Indirubin	10177	479-41-4	Indigo Red	CDK inhibitor / Glycogen synthase kinase inhibitor
JNJ-7706621	5330790	443797-96-4	-----------	CDK inhibitor
Kenpaullone	3820	142273-20-9	NSC 664704	CDK inhibitor / Glycogen synthase kinase inhibitor
PHA-793887	46191454	718630-59-2	-----------	CDK inhibitor
Thiostrepton	16129666	1393-48-2	Bryamycin	FOXM1 inhibitor / Protein synthesis inhibitor
Piceatannol	667639	667639	Hydroxyresveratol	SYK inhibitor
Bortezomib	387447	179324-69-7	Velcade	NFkB pathway inhibitor / Proteasome inhibitor
caffeic-acid-phenethyl-ester	5281787	115610-29-2	Phenethyl caffeate	HIV integrase inhibitor
pyrrolidine-dithiocarbamate	65351	25769-03-3	PDTC	NFkB pathway inhibitor
Triptolide	107985	38748-32-2	PG490	RNA polymerase inhibitor
Cladribine	20279	4291-63-8	Leustatin	Adenosine deamise inhibitor / Ribonucleotide reductase inhibitor

Gemcitabine	60750	95058-81-4	--------------	Ribonucleotide reductase inhibitor
Amonafide	50515	69408-81-7	Nafidimide	Topoisomerase inhibitor
Amsacrine	2179	51264-14-3	mAMSA	Topoisomerase inhibitor
Daurunobicin	30323	20830-81-3	----------------	RNA synthesis inhibitor / Topoisomerase inhibitor
Doxorubicin	31703	23214-92-8	Adriamycin	Topoisomerase inhibitor
Idarubicin	42890	58957-92-9	----------------	Topoisomerase inhibitor
Mitoxantrone	4212	65271-80-9	DHAQ	Topoisomerase inhibitor
Pirarubicin	11296583	72496-41-4	THP-ADM	Topoisomerase inhibitor
Podophyllotoxin	10607	518-28-5	Podofilox	Microtubule inhibitor / Tubulin polymerization inhibitor
Teniposide	452548	29767-20-2	VM-26	Topoisomerase inhibitor
Pifithrin-mu	327653	64984-31-2	2-Phenylethynesulfonamide	HSP inhibitor

Data retrieved from Pubchem (https://pubchem.ncbi.nlm.nih.gov/, accessed in September, 2023). CAS: Chemical Abstracts Service; moa: mechanism of action. * the synonyms provided here are not the only ones available in the database.

[1] Liu, C. J., Hu, F. F., Xia, M. X., Han, L., Zhang, Q., and Guo, A. Y. (2018) GSCALite: a web server for gene set cancer analysis, *Bioinformatics 34*, 3771-3772.
